# Rupture of an Umbilical Hernia, with Prolapsus of Intestines

**Published:** 1882-10

**Authors:** 


RUPTURE OF AN UMBILICAL HERNIA, WITH PROLAPSUS OF INTESTINES.

A farmer's wife, fifty years of age, has had for the last ten years an umbilical hernia which was as large as two fists. She wore a truss, but this consisted, when seen at the accident, of a hard flat pad, without any leather covering, and attached by a common spring. After she had applied the truss she slipped and fell to the ground, with the abdomen forward, and in this accident the walls of the abdomen ruptured, and the intestines prolapsed in large masses. It was three hours after the accident before the first physician arrived. The umbilical region was covered with intestines, which were of a rosy color and but slightly inflated, the omentum not presenting. The wound was about twelve cm. long; it was serrated and did not bleed. The walls of the ruptured abdomen were hardly two to three mm. thick, without any lamellæ distinguishable. The patient did not complain of any pain, and was entirely conscious. Pulse and respiration were normal; no vomiting. The attempted reposition was not successful at first. There was no incarceration, and three fingers could enter into the peritoneal cavity, but reposition was only effected after several incisions had been made into the umbilicus. The wound was closed by silk sutures, and a flannel bandage tightly fixed around the abdomen. The woman died after twenty-four hours. The immediate cause of death was the reduced temperature of the intestines, which had been laid bare for more than three hours.—*Medic. Chirurg. Centralblatt.*

